# Data on PRRSV infection promoted the subtype of porcine dendritic cells from mDCs to pDCs in vivo

**DOI:** 10.1016/j.dib.2016.08.054

**Published:** 2016-09-01

**Authors:** Jinling Liu, Shu Wei, Lixia Liu, Lihui Yu, Chunyan Wang, Yujun Zhao, Fengping Shan, Guoshun Shen

**Affiliations:** a Key Laboratory of Zoonosis of Liaoning Province, College of Animal Science & Veterinary Medicine, Shenyang Agricultural University, Shenyang 110866, P.R. China; b The Preventive Center of Animal Disease of LiaoNing Province, No.95, Renhe Road, Shenbei District, Shenyang 110164, P.R. China; c The 705 hospital Shen Xiang, Shenyang 110016, P.R. China; d Department of Immunology, Basic School of Medicine, China Medical University, No. 92, North Second Road, Shenyang 110001, P.R. China

**Keywords:** PRRSV, Subtype, Dendritic cells

## Abstract

The related study has confirmed that porcine reproductive and respiratory syndrome virus (PRRSV) infection may impair mature states of DCs can lead to suboptimal adaptive immune response (“The role of porcine reproductive and respiratory syndrome virus infection in immunephenotype and Th1/Th2 balance of dendritic cells” (Jinling Liu, We Shu, Liaxia Liu et al., 2016) [1]). In this data article,the porcine dendritic cells (DCs) isolated from porcine peripheral blood and spleen were collected after infection with PRRSV, then the characteristics of the subset differentiation of DCs were analyzed with FACS Calibur Cytometer and fluorescence microscope respectively. This data is an important foundation for further investigation into immune suppression by PRRSV infection, and that, it also provides new data for the development of potential antiviral therapies based on DCs and PRRS vaccines.

**Specifications Table**

**Table:** 

Subject area	*Biology*
More specific subject area	*Microbiology and Immunity*
Type of data	*Figure*
How data was acquired	*Microscope (Thermo) and FACS Calibur Cytometer (Becton-Dickinson Biosciences, San Jose, CA)*
Data format	*Analyzed*
Experimental factors	*Collected pigs infected with PRRSV*
Experimental features	*The distribution of DCs subsets was observed by flow cytometry and fluorescence microscopy in the blood and spleen of infected PRRSV pigs.*
Data source location	*Liaoning Province*
Data accessibility	*Data is within this article*

**Value of the data**

• The data demonstrates that PRRSV infection could lead to adequate humoral immunity by predominantly polarizing pDCs and further supports our publication [1].
• This dataset provides new insights into the mechanism of immune suppression and persistent infection for PRRSV.
• The data is very important as a basis to further clarify the potential antiviral therapies based on DCs.

## 1 Data

**Fig. 1:**
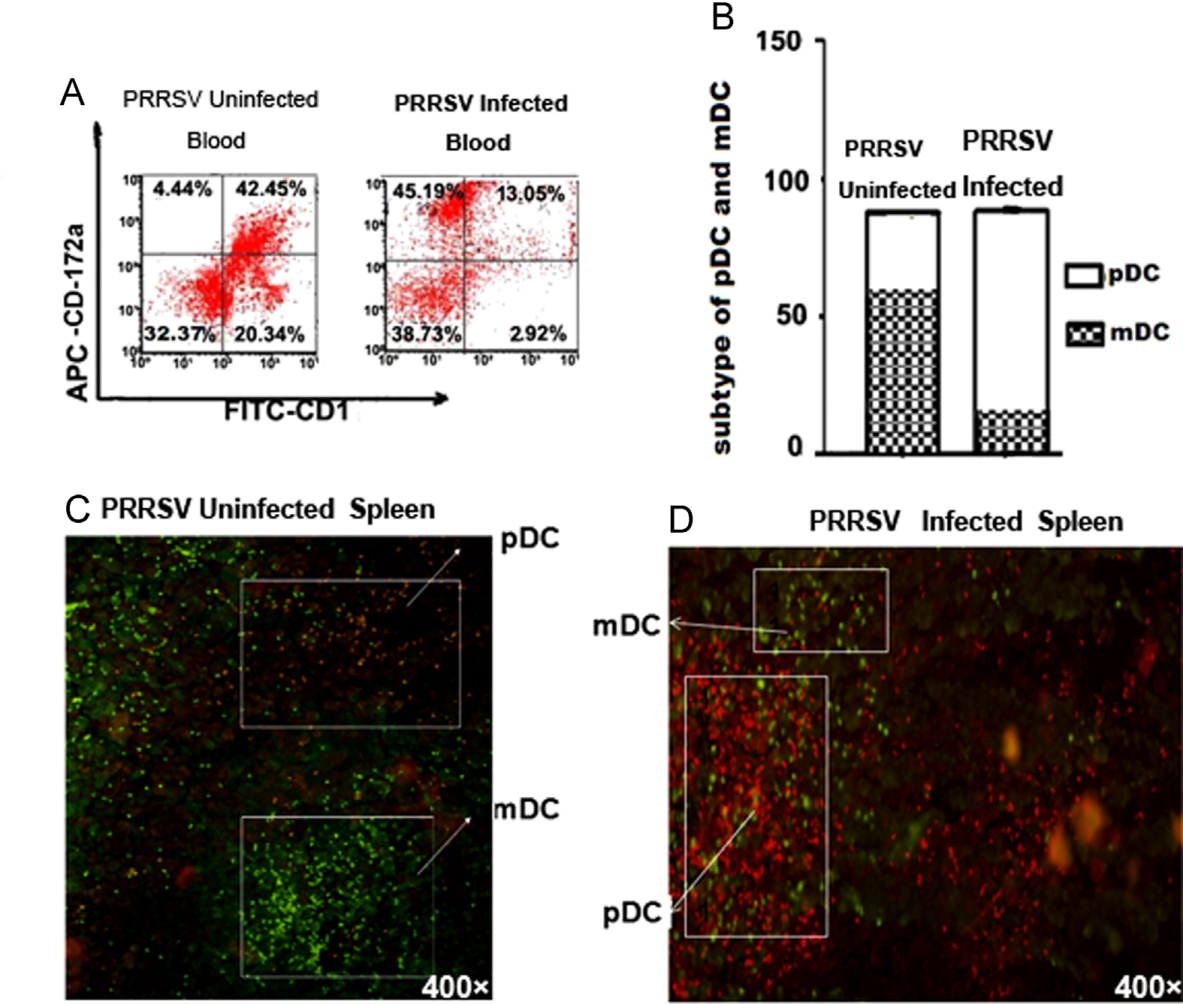
The distribution of DCs subsets. (A) The distribution of mDC and pDC subsets identified with FCM for porcine blood. (B) Statistical graph of mDC and pDC subsets for porcine blood. (C-D). The distribution of mDC and pDC subsets identified with fluorescence microscopy for porcine spleen.

We isolated the porcine peripheral blood and spleen of infected with PRRSV identified by RT-PCR and ELISA, subsets characteristics of DCs were assessed in vivo by flow cytometry (FCM) and fluorescence microscope respectively based on the key surface molecules for pDCs and mDCs. The analyzed data was presented in Fig. 1 and contained two types of data. DCs subtype analysis of porcine peripheral blood (Fig. 1a–b). DCs subtype analysis in porcine spleen (Fig. 1c–d).

## 2 Experimental design, materials and methods

### 2.1 Materials and methods

#### 2.1.1 Screening experimental animals

According to the standard Ministry of Agriculture of China, the experiment pigs were obtained from healthy pigs free from all major diseases except for porcine reproductive and respiratory syndrome virus identified by RT-PCR and ELISA. After that, 4–10 week old piglets were conventionally reared, mixed breed [2]. They were housed in isolation rooms at the Animal Disease Center of LiaoNing Province under the approval of the Institutional Animal Care and Use Committee.

### 2.2 Analysis of DCs subtype in vivo under PRRSV infection

We aseptically collected the porcine peripheral blood from them with heparin anticoagulant (100 μg/mL). Briefly, peripheral blood mononuclear cells (PBMC) were collected from blood by isolating the buffy coat after density centrifugation with lymphocyte-separating medium (Tianjin Hao Yang Biotechnology Company, China). CD14^+^ monocytes were isolated by positive selection of anti-CD14 immunomagnetic beads according to the manufacture׳s protocol (Miltenyi Biotec, Auburn, CA) [3], [4], [5]. Purified CD14^+^ monocytes were re-suspended in PBS at a concentration of 1×10^6^ cells/mL and stained with 1 μL of each of anti-CD1–fluorescein isothiocyanate (FITC) and anti-CD172a allophycocyanin (APC). Afterwards, they were incubated in the dark at 4 °C for 30 min. The suspensions were then washed twice with PBS and the expressions of phenotype molecules mDCs and pDCs were analyzed with FACS Calibur Cytometer (Becton-Dickinson Biosciences, San Jose, CA) and fluorescence microscope [1]. The monocytes isolated from the healthy pigs free from all major diseases were uninfected PRRSV group (mock).

In addition, we prepared slices with the spleen tissue and the distribution of DCs subsets was examined by fluorescence microscopy after staining with specific molecular markers of mDCs and pDCs.
