# Supplementary material for: Data on PRRSV infection promoted the subtype of porcine dendritic cells from mDCs to pDCs in vivo
Source: Data Brief. 2016 Sep 1;9:155–8. doi: 10.1016/j.dib.2016.08.054 (PMC5018077; doi:10.1016/j.dib.2016.08.054)
Supplement: Supplementary file 1 — Supplementary material [file mmc1.doc]

***Conflict of Interest**

No Conflict of Interest.

This study was funded by China Nature Science Project no.31502070 and Dr Start-up fund project of Liaoning no. 20141057. The authors thanks for all researchers who contributed to the work and we apologize to the researchers whose works could not be discussed here due to space limitations.
